# The Role of miR-375-3p and miR-200b-3p in Gastrointestinal Stromal Tumors

**DOI:** 10.3390/ijms21145151

**Published:** 2020-07-21

**Authors:** Ugne Gyvyte, Rokas Lukosevicius, Ruta Inciuraite, Greta Streleckiene, Greta Gudoityte, Justina Bekampyte, Serena Valentini, Violeta Salteniene, Paulius Ruzgys, Saulius Satkauskas, Kristina Zviniene, Juozas Kupcinskas, Jurgita Skieceviciene

**Affiliations:** 1 Institute for Digestive Research, Lithuanian University of Health Sciences, LT-50161 Kaunas, Lithuania; ugne.gyvyte@lsmuni.lt (U.G.); rokas.lukosevicius@lsmuni.lt (R.L.); ruta.inciuraite@lsmuni.lt (R.I.); greta.streleckiene@lsmuni.lt (G.S.); greta.gudoityte@lsmuni.lt (G.G.); justina.bekampyte@lsmuni.lt (J.B.); violeta.salteniene@lsmuni.lt (V.S.); juozas.kupcinskas@lsmuni.lt (J.K.); 2 Department of Pharmacy and Biotechnology (FaBiT), University of Bologna, IT-40126 Bologna, Italy; serena.valentini8@unibo.it; 3 Biophysical Research Group, Faculty of Natural Sciences, Vytautas Magnus University, LT-44404 Kaunas, Lithuania; paulius.ruzgys@vdu.lt (P.R.); saulius.satkauskas@vdu.lt (S.S.); 4 Department of Radiology, Lithuanian University of Health Sciences, LT-50161 Kaunas, Lithuania; kristina.zviniene@lsmuni.lt; 5 Department of Gastroenterology, Lithuanian University of Health Sciences, LT-50161 Kaunas, Lithuania

**Keywords:** gastrointestinal stromal tumor, GIST, miRNA, miR-375-3p, miR-200b-3p

## Abstract

Deregulated microRNA (miRNA) expression profiles and their contribution to carcinogenesis have been observed in virtually all types of human cancer. However, their role in the pathogenesis of rare mesenchymal gastrointestinal stromal tumors (GISTs) is not well defined, yet. In this study, we aimed to investigate the role of two miRNAs strongly downregulated in GIST—miR-375-3p and miR-200b-3p—in the pathogenesis of GIST. To achieve this, miRNA mimics were transfected into GIST-T1 cells and changes in the potential target gene mRNA and protein expression, as well as alterations in cell viability, migration, apoptotic cell counts and direct miRNA–target interaction, were evaluated. Results revealed that overexpression of miR-375-3p downregulated the expression of KIT mRNA and protein by direct binding to KIT 3′UTR, reduced GIST cell viability and migration rates. MiR-200b-3p lowered expression of ETV1 protein, directly targeted and lowered expression of EGFR mRNA and protein, and negatively affected cell migration rates. To conclude, the present study identified that miR-375-3p and miR-200b-3p have a tumor-suppressive role in GIST.

## 1. Introduction

Gastrointestinal stromal tumors (GISTs), while relatively rare, are the most common mesenchymal tumors of the gastrointestinal tract. GISTs are considered to originate from interstitial cells of Cajal or their precursors, residing within the muscle layers of the gastrointestinal tract and are characterized by strong immunohistochemical staining for receptor tyrosine kinase (RTK) KIT (> 95% of the cases) [1,2]. The main initiating events in GIST are gain-of-function mutations in two oncogenes from the RTK family—KIT (70–80%) or platelet-derived growth factor receptor-α (PDGFRA; 10%)—that result in constitutive activation of the receptor and downstream signaling pathways, including mitogen-activated protein kinase (MAPK), phosphatidylinositol 3-kinase (PI3K)/mammalian target of rapamycin (mTOR) or Janus kinase (JAK)/signal transducer and activator of transcription (STAT) [3,4], whereas the lately discovered ETS variant transcription factor 1 (ETV1) is required for the growth and survival of GIST cells [5]. Targeting mutant RTKs with RTK inhibitors are effective in patients with advanced tumors; however, a large proportion of the patients acquire resistance to the treatment in the long run [6], urging the need for the search of new treatment methods and therapeutic targets. Although the main aspects and initial events of GIST biology are already well understood, little is known about the mechanisms underlying the regulation of oncogene expression signatures.

MicroRNAs (miRNAs) are a class of small (22 nucleotides long), highly stable non-coding RNAs involved in post-transcriptional regulation of gene expression [7]. Deregulated miRNA expression profiles have been observed in virtually all major types of cancer, where they can act both as oncogenes or tumor suppressors and are associated with tumorigenesis, tumor progression, metastasis and drug resistance pathways [7,8,9,10]. Furthermore, miRNAs have been shown to exhibit a diagnostic or prognostic value and even have potential clinical implications for targeted gene therapy in cancer patients [11,12,13]. Several miRNA profiling and functional studies have shown the importance of miRNAs in GIST [14,15]. However, studies characterizing miRNA target genes and their molecular mechanism of action in GIST are still lacking.

In this study, we aimed to get more insight into the potential role of two miRNAs (miR-375-3p and miR-200b-3p) that were highly downregulated in GIST in our previous miRNA profiling study [15] and were predicted to target GIST- and oncogenic signaling-related genes in silico. We evaluated how overexpression of these miRNAs affect target gene and protein expression, as well as viability, migration and apoptosis of GIST representing cells, and confirmed the tumor-suppressive role of miR-375-3p and miR-200b-3p through direct target regulation. We believe that these findings reveal the important role of miR-375-3p and miR-200b-3p in GIST tumorigenesis and their therapeutic potential.

## 2. Results

### 2.1. Overexpression of miR-375-3p and miR-200b-3p Alters the Expression of Their Putative Target Genes

**Figure 1:**
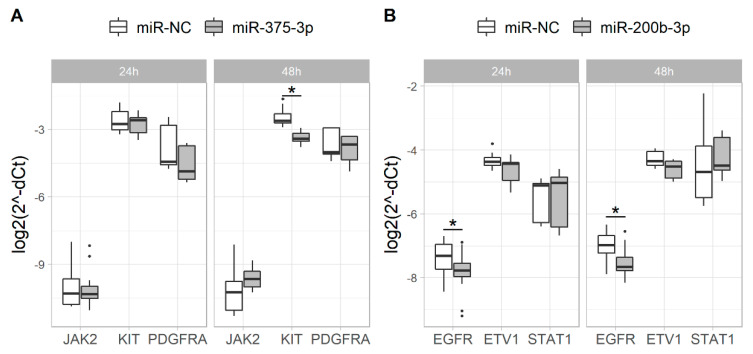
Effect of (**A**) miR-375-3p and (**B**) miR-200b-3p overexpression to target gene mRNA expression in GIST-T1 cells compared to gene expression in cells transfected with a mimic negative control (miR-NC) measured 24 h and 48 h after transfection. Gene expression was normalized to the expression values of the *GAPDH* reference gene. Data from three to five independent experiments each containing three biological replicates. * *p* < 0.05; middle line in the box—median value; whiskers—min. and max. values.

**Figure 2:**
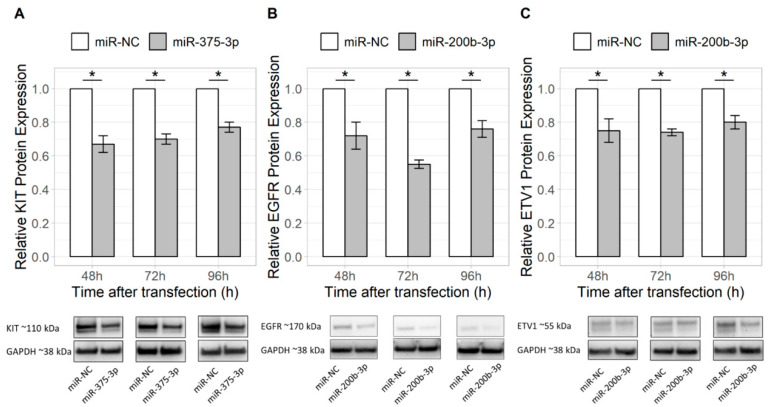
Effect of miR-375-3p and miR-200b-3p overexpression to target protein expression in GIST-T1 cells compared to protein expression in cells transfected with a mimic negative control (miR-NC) measured 48 h, 72 h and 96 h after transfection. (**A**) Effect of miR-375 on KIT protein, (**B**) miR-200b-3p on EGFR protein and (**C**) miR-200b-3p in ETV1 protein. Protein bands representing the signals detected by Western blot are provided at the bottom of the figure. Protein expression was normalized to the expression values of GAPDH reference protein. Data from three to five independent experiments. * *p* < 0.05.

Using in silico prediction tools *KIT*, *PDGFRA* and *JAK2* were selected as potential target genes for miR-375-3p, and *EGFR*, *ETV1* and *STAT1* were selected as targets for miR-200b-3p. Upregulation of miR-375-3p reduced expression of *KIT* mRNA (48 h after transfection, *p* = 1.289 × 10^−8^; Figure 1) and protein (48 h, 72 h and 96 h after transfection, *p* = 0.020, *p* = 0.003 and *p* = 0.003, respectively; Figure 2) in the GIST-T1 cell line, compared to the mimic negative control. Overexpressed miR-200b-3p significantly reduced expression of *EGFR* mRNA (24 h and 48 h after transfection, *p* = 0.016 and *p* = 0.004, respectively; Figure 1), as well as EGFR (48 h, 72 h and 96 h after transfection, *p* = 0.021, *p* = 0.003 and *p* = 0.001, respectively) and ETV1 (48 h, 72 h and 96 h after transfection, *p* = 0.021, *p* = 0.021 and *p* = 0.003, respectively) proteins (Figure 2). No changes in expression of JAK2 and PDGFRA, as well as STAT1, were observed after transfection with miR-375-3p or miR-200b-3p mimics, respectively.

### 2.2. miR-375-3p and miR-200b-3p Directly Regulate Their Predicted Targets KIT and EGFR

**Figure 3:**
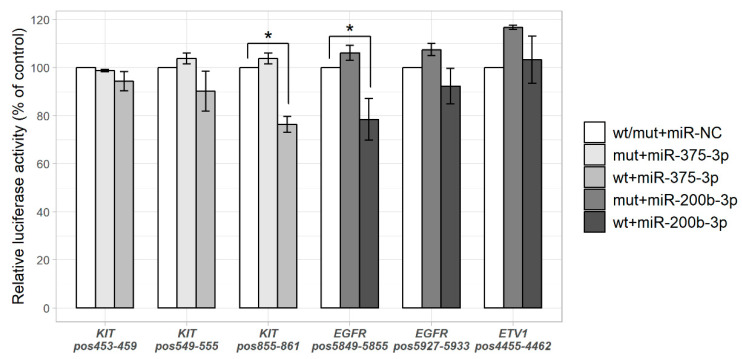
Estimation of direct miRNA-target interaction by luciferase reporter assay. GIST-T1 cells were cotransfected with miRNA mimic (or miRNA mimic negative control) and pmiR-REPORT luciferase vector, containing wild-type (wt) or mutant (mut) 3′UTR sequences of the predicted target genes. Several different miRNA binding positions (pos) in the predicted target gene were investigated (Tables S1 and S2). Luciferase activity was normalized to the β-galactosidase signals. Results are shown as a percentage relative to the mimic negative control (miR-NC). Data from three to five independent experiments. * *p* < 0.05.

Direct binding of miR-375-3p to KIT and miR-200b-3p to EGFR and ETV1 was evaluated using luciferase reporter system containing 3′ UTR-wild type and 3′ UTR-mutant regions of the genes. Cells were co-transfected with the mimic of interest (miR-375-3p, miR-200b-3p or miRNA mimic negative control) and the reporter vector. The results indicated that miR-375-3p significantly reduced firefly luciferase activity in *KIT*-3′UTR-wt (*p* = 0.007) and miR-200b-3p—in EGFR-3′UTR-wt (*p* = 0.020, compared to the negative control; Figure 3), indicating a direct miRNA–target interaction. Firefly luciferase activity did not change in cells transfected with the mut-type vectors.

### 2.3. miR-375-3p Reduced Cell Viability and Proliferation

**Figure 4:**
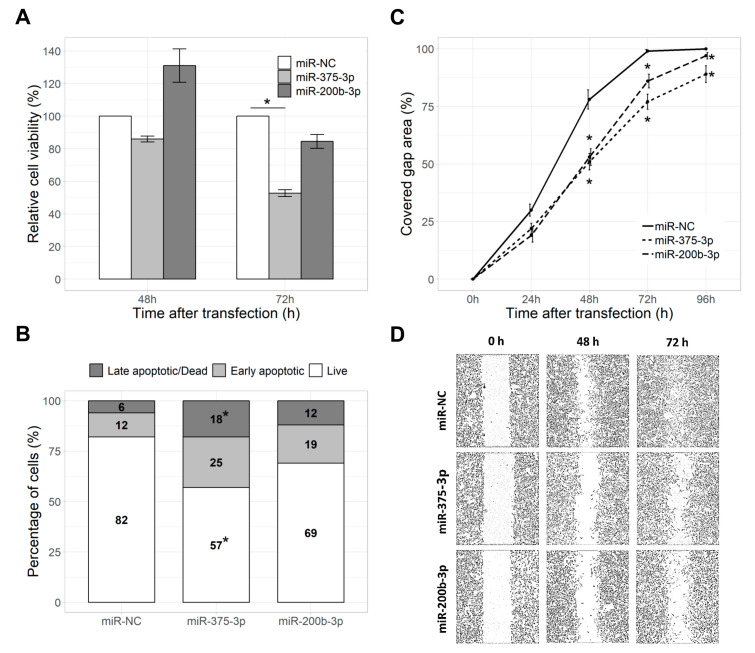
Effect of miR-375-3p and miR-200b-3p on cell viability, migration and apoptosis: (**A**) Barplot represents changes in cell viability 48 h and 72 h after transfection with miRNA mimics relative to cells transfected with mimic negative control, (**B**) stacked barplot represents the percentage of live, early apoptotic or late apoptotic/dead cells 72 h after transfection with miRNA mimics and (**C**) line plot shows cell migration rates, represented as the percentage of the covered gap area, measured 0–96 h after transfection with miRNA mimics. (**D**) Microscopic images of the closing gap in the wound healing assay illustrating differences in the cell migration rates after transfection with miRNA mimics. Data from three to five independent experiments. *—*p* < 0.05.

To evaluate the effect of miR-375-3p and miR-200b-3p on GIST-T1 cell viability and proliferation, the MTT assay was performed after transfection with respective miRNA mimics. miR-375-3p significantly reduced cell viability by 47% 72 h after transfection (*p* = 0.029), compared to the mimic negative control (Figure 4A). Overexpression of miR-200b-3p had no significant effect on the viability and proliferation of GIST-T1 cells.

### 2.4. miR-375-3p and miR-200b-3p Reduced Cell Migration Rate

Reduced GIST-T1 cell migration rate was observed after transfection with both miR-375-3p and miR-200b-3p in the Wound Healing Assay. Cells affected with miRNA mimic negative control nearly fully covered the gap 72 h after transfection (coverage of 99%) and reached full gap coverage 96 h after transfection (100%). Overexpression of miR-375-3p resulted in significantly slower gap closure 48 h (by 28%, *p* = 0.001), 72 h (by 23%, *p* = 0.003) and 96 h (by 11%, *p* = 0.002) after transfection, compared to the mimic negative control. miR-200b-3p significantly slowed the gap closure 48 h, 72 h and 96 h after transfection, differing from the mimic negative control by 25% (*p* = 0.003), 13% (*p* = 0.012) and 3% (*p* = 0.002), respectively (Figure 4C,D).

### 2.5. miR-375-3p and miR-200b-3p Did Not Affect Cell Apoptosis

To investigate changes in the rates of early apoptosis and cell death, a flow-cytometry-based Annexin V-FITC/PI assay was employed, where annexin V-FITC-positive cells were considered as early apoptotic and annexin V-FITC/PI-positive—as late apoptotic/necrotic cells. Overexpression of miR-375-3p resulted in a significantly lower amount of live cells (by 25%, *p* = 0.029) and a higher number of late apoptotic/necrotic cells (by 12%, *p* = 0.029), while the amount of early apoptotic cells was increased by 13%, but the result was not significant. Transfection with the miR-200b-3p mimic only slightly altered numbers of live, early apoptotic or late apoptotic/necrotic cells, but the differences were not significant (Figure 4B).

## 3. Discussion

Although miRNAs have been extensively studied as important regulators of cellular processes contributing to the development of a variety of human cancers and an increasing number of studies reveal their role in GIST [14], the involvement of specific miRNAs in the pathology of these rare tumors, their targets and functions remain scarcely investigated. In this study, we employed data from our previous miRNA profiling study [15] and selected two miRNAs highly downregulated in GIST tissue—miR-375-3p and miR-200b-3p—for the further investigation of their functional role in GIST representing cell line. We determined that miR-375-3p and miR-200b-3p directly regulated genes involved in GIST tumorigenesis and their overexpression had a tumor-suppressive role.

miR-375-3p has been previously described as a tumor suppressor frequently downregulated in multiple types of cancer including gastric cancer, hepatocellular carcinoma, head and neck cancer and having a potential as a diagnostic and prognostic biomarker [16]. To our best knowledge, this miRNA has not been investigated in GIST before. In this study, we found that the upregulation of miR-375-3p reduced GIST cell viability and migration rates. In line with our results, it has been previously reported that miR-375-3p reduced cell viability in liver cancer cells [17], inhibited migration of pancreatic cancer cells [18], suppressed cell proliferation in colorectal carcinoma [19], affected migration, invasion and epithelial–mesenchymal transition in gastric cancer cells [20,21] through regulation of different targets. Additionally, our data revealed that overexpression of miR-375-3p resulted in the downregulation of KIT mRNA and protein levels. A luciferase reporter assay showed the regulation of luciferase signal by miR-375-3p through the KIT 3′UTR, confirming that miR-375-3p directly targets KIT. The tyrosine kinase receptor KIT was predicted as one of the potential targets of miR-375-3p using in silico miRNA-target prediction tool TargetScan and was selected for further investigation due to its essential role in the initiation of GIST tumorigenesis. KIT protein overexpression was observed in the majority of GIST cases, while gain-of-function mutations in the KIT gene lead to activation of downstream signaling pathways, including MAPK, PI3K-AKT-mTOR, JAK-STAT and result in promoted tumor growth and inhibition of apoptosis [22,23,24,25], making KIT an attractive target for cancer therapy. However, despite the existing targeted therapy by tyrosine kinase inhibitors applied in advanced GISTs, many patients eventually acquire resistance to the treatment or suffer from severe side effects caused by second-line inhibitors [2]. Therefore, induced overexpression of miR-375-3p in GIST could significantly reduce KIT levels and might be a promising tool for GIST treatment, worth further investigation.

In addition, miR-375-3p has been shown to be implicated in gastric cancer through interaction with the components of the JAK-STAT pathway [26,27], while JAK2 blockade led to tumor growth inhibition and apoptosis in GIST [28]. However, in this study, overexpression of miR-375-3p did not affect expression levels of JAK2 mRNA in GIST cells and this interaction was not further investigated.

MiR-200b-3p belongs to a miR-200 miRNA family, known to be associated with a wide range of human cancers [29], and has been mainly reported as a tumor suppressor inhibiting cell growth and motility of breast cancer cells [30], suppressing metastasis in renal cell carcinoma and breast cancer [31,32], inhibiting proliferation, cell cycle and inducing apoptosis in gastric and colorectal cancers [33,34]. Nevertheless, the role of miR-200b-3p in cancer remains controversial, since studies showing the opposite effects on cancer cells exist [35,36]. Here we found that miR-200b-3p negatively affected cell migration rates and lowered expression of ETV1 and EGFR proteins in GIST cells. ETV1 is another important player in GIST pathogenesis, which is stabilized by KIT and acts as a master regulator of GIST-specific transcription network, promoting tumorigenesis [5]. Although miR-200b-3p lowered expression of ETV1, the direct miRNA–target interaction was not confirmed and downregulation of ETV1 was possibly an indirect effect mediated through additional targets, leaving the underlying genes and molecular mechanisms unclear. Nevertheless, our results revealed a direct binding of miR-200b-3p to EGFR 3′UTR. EGFR is a tyrosine kinase, often mutated, overexpressed and acting as a promoter of tumorigenesis in epithelial tumors, such as lung, breast cancer and glioblastoma [37]; however, the role of EGFR in GIST is not that well-investigated and yet controversial. EGFR and its ligands have been shown to be expressed in the majority of GISTs and is known to canonically activate downstream pathways, including RAS/MAPK and PI3K/AKT [37], that are also overrepresented in GIST [22]. Although expression of EGFR was not associated with the malignant GIST phenotype [38] and was more likely to be an indicator of favorable prognosis in gastric GIST [39], combined targeting of EGFR and KIT increased efficiency of the treatment and abrogated resistance to tyrosine kinase inhibitor imatinib [40], revealing the importance and potential of EGFR as a therapeutic target. Therefore, even though miR-200b-3p alone might not have remarkable effects inhibiting GIST, it could possibly be of value as a component for combined therapy strategies.

This study has certain limitations that need to be acknowledged. The main drawback is that the evaluation of the functional role of investigated miRNAs was performed only in one GIST representing cell line. Although different GIST cell lines with varying characteristics exist (GIST-882, GIST-T1-R, GIST-48, etc.), only one (GIST-T1) is commercially available. Nevertheless, GIST-T1 cells express all important GIST biomarkers and fully represent characteristics of GIST [41]. Additionally, the effect of target gene silencing on GIST-T1 cells was not evaluated in this study. However, the investigated targets and consequences of their inhibition in GIST have been previously described in other studies [40,42].

In conclusion, the present study showed that the upregulation of miR-375-3p and miR-200b-3p exhibit antitumor effects in GIST representing cells via downregulation of GIST-related targets. Therefore, these miRNAs might play a significant role in the pathogenesis of GIST and could be promising candidates for molecular therapy, based on the enhanced expression of miRNAs.

## 4. Materials and Methods

### 4.1. Cell Culture

Commercial GIST-T1 cell line (cat. no.: PMC-GIST01-COS), derived from GIST of the stomach of a Japanese woman [41], was acquired from Cosmo Bio Co., LTD (Tokyo, Japan). Cells were cultured in RPMI 1640 GlutaMAX^TM^ cell culture media supplemented with 10% of fetal bovine serum (Gibco^TM^, Thermo Fisher Scientific, Waltham, MA, USA) and 1% of penicillin–streptomycin (Corning^®^, New York, NY, USA) in a humidified incubator containing 5% CO_2_ at 37 °C. A media without antibiotics was used for transfection experiments. Cell lines were tested for mycoplasma contamination using specific primers [43].

### 4.2. miRNA Selection and Target Prediction

miRNAs that have been found to be strongly deregulated in GIST [15] and have been linked with cancer were involved in this study. Possible target genes were retrieved from the TargetScan database (release 7.0) [44] and selected based on their role in other human cancers (oncogene or tumor-suppressor) and involvement in GIST-related signaling pathways.

### 4.3. Cell Transfection

miRVana miRNA mimics of miR-375-3p (assay ID: MC10327), miR-200b-3p (assay ID: MC10492) and non-specific miRNA Mimic Negative Control #1 (Invitrogen^TM^, Carlsbad, CA, USA) were transfected into cells at a final concentration of 100 nM using Lipofectamine3000^TM^ reagent (Invitrogen^TM^, Carlsbad, CA, USA), 24 h after cell seeding. Measurement time points and optimal concentration of reagents were selected based on manufacturer‘s recommendations, scientific publications [45] and experimental data. The efficiency of transfection and silencing was tested by measuring changes in miRNA expression by qRT-PCR (TaqMan^TM^ MicroRNA Assay IDs 000564 for miR-375-3p and 002251 for miR-200b-3p, Applied Biosystems^TM^, Foster City, CA, USA) as well as using positive transfection control (miRVana miRNA mimic miR-1 Positive Control) and measuring the effect on the expression of its target PTK9 at mRNA (TaqMan^TM^ Gene Expression Assay ID: Hs00911809_g1, Applied Biosystems^TM^, Foster City, CA, USA) and protein levels (1:1000, anti-TWF1, Rabbit Polyclonal, ab154725, Abcam^®^, Cambridge, UK; Figure S1).

### 4.4. Quantitative Reverse Transcription PCR

To estimate target gene mRNA expression, total RNA was extracted from cell lysates (6 × 10^4^ cells/well were seeded in 24-well plates) 24 h and 48 h after transfection with miRNA mimics using RNeasy^®^ Mini Kit (Qiagen, Hilden, Germany). On-column DNase digestion was applied to remove residual DNA using the RNase-free DNase Set (Qiagen, Hilden, Germany). Total RNA was further reverse transcribed using a High-Capacity cDNA Reverse Transcription Kit (Applied Biosystems^TM^, Foster City, CA, USA). Expression levels were measured using TaqMan^TM^ Gene Expression Assays (Assay IDs: *KIT* Hs00174029_m1; *PDGFRA* Hs00998026_m1; *ETV1* Hs00951951_m1; *EGFR* Hs01076090_m1; *STAT1* Hs01013996_m1; *JAK2* Hs10178136) on the 7500 Fast Real-Time PCR System (Applied Biosystems^TM^, Foster City, CA, USA). The expression was normalized to the expression levels of *GAPDH* (Assay ID: Hs99999905_m1) reference gene. All the procedures were performed according to the manufacturer‘s protocol.

### 4.5. Western Blot

In 6-well plates, 3 × 10^5^ cells/well were seeded for protein expression experiments. Cells were lysed using 1× RIPA buffer (Abcam^®^, Cambridge, UK) complemented with a cocktail of proteinase and phosphatase inhibitors (Sigma Aldrich, St. Louis, MO, USA) 48 h, 72 h and 96 h after transfection with miRNA mimics. Protein concentrations were measured using the Pierce BCA Protein Assay Kit (Thermo Scientific, Waltham, MA, USA). Protein lysates were separated on 4-12% Bis-Tris gels and transferred to 0.45 µm PVDF membranes. After blocking with WesternBreeze Blocker/Diluent (Part A and B; Invitrogen^TM^, Carlsbad, CA, USA) at room temperature for 1 h, membranes were incubated with primary antibodies against KIT (1:1000 dilution, rabbit monoclonal, ab32363, Abcam^®^), EGFR (1:1000 dilution, mouse monoclonal, sc-373746, Santa Cruz Biotechnology, Dallas, TX, USA), ETV1 (1:1000, rabbit polyclonal, ab184120, Abcam^®^, Cambridge, UK) and GAPDH (0.4 µg/mL, mouse monoclonal, AM4300, Invitrogen^TM^, Carlsbad, CA, USA) as a loading control overnight at 4 °C. Anti-rabbit and Anti-mouse alkaline-phosphatase conjugated secondary antibody solutions and Novex^TM^ AP Chemiluminescent Substrate (CDP-Star^®^) (Invitrogen^TM^, Carlsbad, CA, USA) were used for protein detection with a BioRad ChemiDoc XRS+ System (BioRad Laboratories, Hercules, CA, USA). Images were analyzed with ImageLab^TM^ software (BioRad Laboratories, Hercules, CA, USA).

### 4.6. Luciferase Reporter Assay

The pMIR-REPORT^TM^ luciferase reporter vectors (pMIR-REPORT^TM^ miRNA Expression Reporter Vector System, Invitrogen^TM^, Carlsbad, CA, USA) were created by cloning fragments corresponding to the 3‘UTR of the KIT, ETV1 and EGFR mRNA (including wild-type and mutant miRNA binding sites), between the HindIII and BcuI sites in the 3′UTR of the firefly luciferase gene, according to the manufacturer’s instructions. The oligonucleotide sequences and predicted miRNA-mRNA pairing schemes are listed in Tables S1 and S2. Constructed vectors were verified by Sanger sequencing using Applied Biosystems^TM^ 3500 analyzer (Applied Biosystems^TM^, Foster City, CA, USA). The human gastric adenocarcinoma cell line AGS, obtained from the American Type Culture Collection (ATCC), was used as a model system for direct miRNA–target gene interaction analysis since these cells are easier to cultivate and transfect. Cells were plated at 1 × 10^5^ cells/well in triplicates in 24-well plates and co-transfected with 146 ng of pMIR-REPORT^TM^ luciferase constructs (wt or mut vector), 29 ng pMIR-REPORT^TM^ β-Galactosidase reporter control vector and 50 nM of either miRNA mimic or mimic negative control using Lipofectamine 3000 (Invitrogen^TM^, Carlsbad, CA, USA). Forty-eight hours after the incubation, luciferase activity was measured using the Dual-Light™ Luciferase and β-Galactosidase Reporter Gene Assay System (Invitrogen^TM^, Carlsbad, CA, USA) on the Tecan GENios Pro microplate reader (Tecan Trading AG, Mannedorf, Switzerland), following the manufacturer’s protocol. Firefly luciferase activity was normalized to β-Galactosidase expression for each sample.

### 4.7. MTT Assay

Viability and proliferation of cells (15 × 10^3^ cells/well) were evaluated using 3-(4,5-dimethylthiazol-2-yl)-2,5-diphenyltetrazolium bromide (MTT) cell proliferation assay 48 h and 72 h after transfection with miRNA mimics. Of the MTT solution 20 µL (final concentration 0.5 mg/mL; ATCC^®^) was added to each well containing GIST-T1 cells and the plate was incubated at 37 °C for 2 h. Formed formazan crystals were dissolved in 200 µL of DMSO (Carl Roth^®^, Karlsruhe, Germany) at 37 °C for 15 min. Optical density values at 570 nm (with a reference filter of 620 nm) were measured using a Sunrise absorbance microplate reader (Tecan Trading AG, Mannedorf, Switzerland).

### 4.8. Apoptosis Assay

Cell apoptosis was measured using FITC Annexin V Apoptosis Detection Kit II (BD Pharmigen^TM^, BD Biosciences, Franklin Lakes, NJ, USA) 72 h after transfection with miRNA mimics, following the manufacturer’s protocol. In short, cells were collected using StemPro^TM^ Accutase^TM^ cell dissociation reagent (Gibco, Thermo Fisher Scientific), centrifuged and resuspended in a 1 × binding buffer at a concentration of 1 × 10^6^ cells/mL. Of cell suspension 100 µL (1 × 10^5^ cells) were incubated with FITC-Annexin V and PI for 15 min at room temperature in the dark. Of binding buffer 400 µL was added before the analysis by flow cytometer (BD Accuri C6, DB Biosciences, Erembodegem, Belgium). Cells were discriminated into live (both annexin V-FITC/PI negative), early apoptotic (annexin V-FITC positive) and late apoptotic/dead cells (both annexin V-FITC/PI-positive).

### 4.9. Wound Healing Assay

The wound healing assay was performed to evaluate cell migration rates under the effect of miRNA mimics. Reverse transfected cells (5 × 10^4^ cells/well) were seeded into the 2-well cell culture insert (iBidi^®^, Martinsried, Planegg, Germany). Twenty-four hours after transfection, the insert was removed to form a 500 µm wide gap. The gap was washed with PBS to remove unattached cells. Pictures of the closing gap were captured every 24 h until the full gap closure using the Olympus^®^ IX71 (Tokyo, Japan) microscope.

### 4.10. Statistical Analysis

All the statistical analyses were performed using R Studio software (version 3.5.2). Data distribution was determined by the Shapiro–Wilk test and differences analyzed by the two-sided Mann–Whitney U (Wilcoxon rank-sum) or Wilcoxon signed-rank test. The difference between the values was considered significant when *p* < 0.05. Experimental data are presented as means ± standard deviation of three to five independent experiments.

## Supplementary Materials

Supplementary materials can be found at https://www.mdpi.com/1422-0067/21/14/5151/s1. Figure S1: Evaluation of miRNA transfection and silencing efficiency. Table S1: Binding positions and insert sequences for the construction of pMIR-REPORT-Luciferase vectors, used for the investigation of direct miRNA-target binding. Table S2: Predicted pairing of target region and miRNA (data obtained from the TargetScanHuman database).

## Abbreviations

3′UTR	three prime untranslated region
ATCC	American type culture collection
DMSO	dimethyl sulfoxide
EGFR	epidermal growth factor receptor
ETV1	ETS transcription factor 1
FITC	fluorescein isothiocyanate
GAPDH	glyceraldehyde 3-phosphate dehydrogenase
GIST	gastrointestinal stromal tumor
JAK	Janus kinase
MAPK	mitogen-activated protein kin
miRNA	microRNA
mTOR	mammalian target of rapamycin
MTT	3-(4,5-dimethylthiazol-2-yl)-2,5-diphenyltetrazolium bromide
mut	mutant
NC	negative control
PDGFRA	platelet-derived growth factor receptor-α
PI	propidium iodide
PI3K	phosphatidylinositol-3-kinase
RIPA	radioimmunoprecipitation assay
RNA	ribonucleic acid
RT-qPCR	Reverse transcription quantitative polymerase chain reaction
RTK	receptor tyrosine kinase
STAT	signal transducer and activator of transcription
wt	wild-type
