# Supplementary material for: The Role of miR-375-3p and miR-200b-3p in Gastrointestinal Stromal Tumors
Source: Int J Mol Sci. 2020 Jul 21;21(14):5151. doi: 10.3390/ijms21145151 (PMC7404198; doi:10.3390/ijms21145151)
Supplement: Supplementary file 1 [file ijms-21-05151-s001.pdf]

Supplementary data

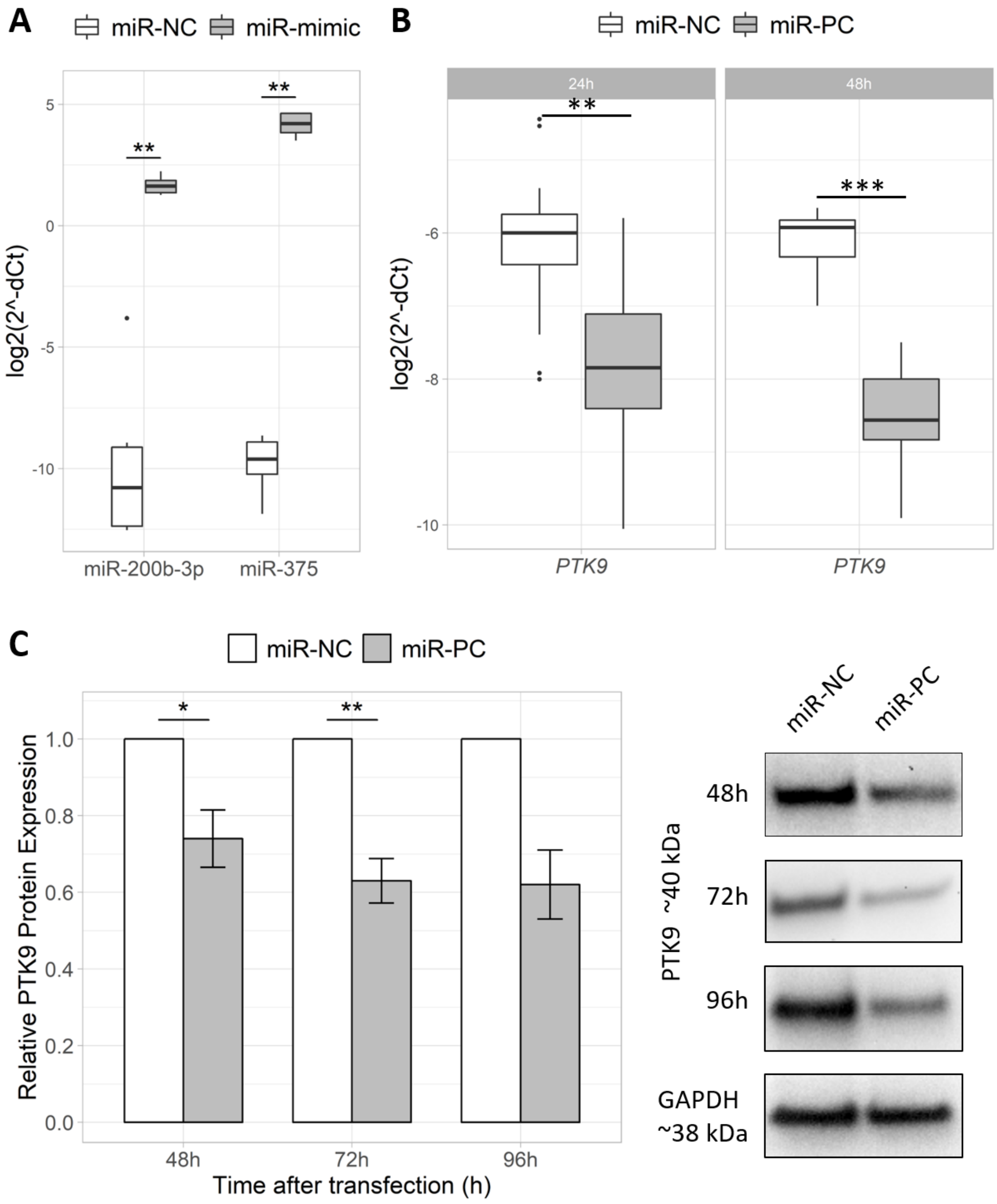


**Figure S1:** Evaluation of miRNA transfection and silencing efficiency. (A) Upregulation of miRNAs miR-375-3p and miR-200b-3p was observed after transfection with corresponding miRNA mimics; (B) PTK9 gene was downregulated after transfection with miRVana miRNA Mimic miR-1 positive control and (C) PTK9 protein was downregulated after transfection with miRVana miRNA Mimic miR-1 positive control. miR-NC—miRNA mimic negative control, miR-PC—miRNA mimic positive control. * $p < 0.05$, ** $p < 0.005$, *** $p < 0.0005$.

**Table S1:** Binding positions and insert sequences for the construction of pMIR-REPORT-Luciferase vectors, used for the investigation of direct miRNA-target binding. The sequences coding the predicted microRNA binding sites in the 3'UTR of the target genes are highlighted in grey. Changed nucleotides in the mutant insert sequences are colored in red.

| Mature miRNA ID | Mature miRNA Accession Number | Target Symbol | Target Ensembl Number | miRNA Binding Position | Insert type | Insert sequences (5' to 3') | |
|---|---|---|---|---|---|---|---|
| **miR-375-3p** | MIMAT 0000728 | ***KIT*** | ENSG 00000157404 | 453-459 | Wt | Forward | CTGCGCACTAGTTGGAAAACAGAACATCATTAGAACAAAGGACAGAGTATGAACACCTGAAGCTTAATAAA |
| | | | | | | Reverse | TTTATTAAGCTTCAGGTGTTCATACTCTGTCCTTTGTTCTAATGATGTTCTGTTTTCCAACTAGTGCGCAG |
| | | | | | Mut | Forward | CTGCGCACTAGTTGGAAAACAGAACATCATTAGATGAAAGGACAGAGTATGAACACCTGAAGCTTAATAAA |
| | | | | | | Reverse | TTTATTAAGCTTCAGGTGTTCATACTCTGTCCTTTCATCTAATGATGTTCTGTTTTCCAACTAGTGCGCAG |
| | | | | 549-555 | Wt | Forward | CTGCGCACTAGTAAAAAATGATCCCCAAGTGTGAACAAAAGATGCTCTTCTGTGGACCAAAGCTTAATAAA |
| | | | | | | Reverse | TTTATTAAGCTTTGGTCCACAGAAGAGCATCTTTTGTTCACACTTGGGGATCATTTTTTACTAGTGCGCAG |
| | | | | | Mut | Forward | CTGCGCACTAGTAAAAAATGATCCCCAAGTGTGAAGGAAAGATGCTCTTCTGTGGACCAAAGCTTAATAAA |
| | | | | | | Reverse | TTTATTAAGCTTTGGTCCACAGAAGAGCATCTTTCCTTCACACTTGGGGATCATTTTTTACTAGTGCGCAG |
| | | | | 855-861 | Wt | Forward | CTGCGCACTAGTATAACCACAAAGCACAGTTTGAACAAAATCTCCTCTTTTAGCTGATGAAGCTTAATAAA |
| | | | | | | Reverse | TTTATTAAGCTTCATCAGCTAAAAGAGGAGATTTTGTTCAAACTGTGCTTTGTGGTTATACTAGTGCGCAG |
| | | | | | Mut | Forward | CTGCGCACTAGTATAACCACAAAGCACAGTTTGAAGGAAATCTCCTCTTTTAGCTGATGAAGCTTAATAAA |
| | | | | | | Reverse | TTTATTAAGCTTCATCAGCTAAAAGAGGAGATTTCCTTCAAACTGTGCTTTGTGGTTATACTAGTGCGCAG |
| **miR-200b-3p** | MIMAT 0000318 | ***EGFR*** | ENSG 00000146648 | 5849-5855 | Wt | Forward | TGCGCACTAGTAAGTTTTGCAATGATGAAAGCAGTATTTGTACAAATGAAAAGCAGAAAAGCTTAATAA |
| | | | | | | Reverse | TTATTAAGCTTTTCTGCTTTTCATTTGTACAAATACTGCTTTCATCATTGCAAAACTTACTAGTGCGCA |
| | | | | | Mut | Forward | CTGCGCACTAGTAAGTTTTGCAATGATGAAAGCAGCCTTTGTACAAATGAAAAGCAGAAAAGCTTAATAAA |
| | | | | | | Reverse | TTTATTAAGCTTTTCTGCTTTTCATTTGTACAAAGGCTGCTTTCATCATTGCAAAACTTACTAGTGCGCAG |
| | | | | 5927-5933 | Wt | Forward | TGCGCACTAGTGAAATGTTGATTGTGCATTGAGTATTAAAAAAATTAGATGTATATTATAAGCTTAATAA |
| | | | | | | Reverse | TTATTAAGCTTATAATATACATCTAATTTTTTAATACTCAATGCACAATCAACATTTCACTAGTGCGCA |
| | | | | | Mut | Forward | CTGCGCACTAGTGAAATGTTGATTGTGCATTGAGTCCTAAAAAAATTAGATGTATATTATAAGCTTAATAAA |
| | | | | | | Reverse | TTTATTAAGCTTATAATATACATCTAATTTTTTAGGACTCAATGCACAATCAACATTTCACTAGTGCGCAG |
| | | ***ETV1*** | ENSG 00000006468 | 4455-4462 | Wt | Forward | CTGCGCACTAGTCTTTTTTGCATATAGACTCCAGTATTAGTTACTTTCATTCTAAAATTAAGCTTAATAAA |
| | | | | | | Reverse | TTTATTAAGCTTAATTTTAGAATGAAAGTAACTAATACTGGAGTCTATATGCAAAAAAGACTAGTGCGCAG |
| | | | | | Mut | Forward | CTGCGCACTAGTCTTTTTTGCATATAGACTCCAGGCTTAGTTACTTTCATTCTAAAATTAAGCTTAATAAA |
| | | | | | | Reverse | TTTATTAAGCTTAATTTTAGAATGAAAGTAACTAAGCCTGGAGTCTATATGCAAAAAAGACTAGTGCGCAG |

**Table S2.** Predicted pairing of target region and miRNA (data obtained from the TargetScanHuman database). The microRNA-target binding sites are highlighted in grey. Changed nucleotides in the mutant insert sequences are colored in red.

| | Predicted pairing of target region (top) and miRNA (bottom) | |
|---|---|---|
| | Wild type target sequence | Mutant target sequence |
| Position 453-459 of KIT 3' UTR<br><br>hsa-miR-375 | 5' ...AACAGAACAUCAUUAGAACAAAG...<br>\|\| \| \| \|\|\|<br>3' AGUGCGCUCGGCUUGCUUGUUU | 5' ...AACAGAACAUCAUUAGATGAAAG...<br>\|\| \|\|<br>3' AGUGCGCUCGGCUUGCUUGUUU |

| | | |
|---|---|---|
| Position 549-555 of KIT 3' UTR<br><br>hsa-miR-375 | 5' ...AUGAUCCCCAAGUGUGAACAAAA...<br>\|\| \|\| \|\|<br>3' AGUGCGCUCGGCUUGCUUGUUU | 5' ...AUGAUCCCCAAGUGUGAAGGAAA...<br>\|\| \| \|<br>3' AGUGCGCUCGGCUUGCUUGUUU |
| Position 855-861 of KIT 3' UTR<br><br>hsa-miR-375 | 5' ...CACAAAGCACAGUUUGAACAAAA...<br>\| \|\| \| \| \|\| \|\|<br>3' AGUGCGCUCGGCUUG-- CUUGUUU | 5' ...CACAAAGCACAGUUUGAAGGAAA...<br>\| \|\| \| \| \| \|<br>3' AGUGCGCUCGGCUUG-- CUUGUUU |
| Position 5849-5855 of EGFR 3' UTR<br><br>hsa-miR-200b-3p | 5' ...UUGCAAUGAUGAAAGCAGUAUUU...<br>\| \|\| \|\| \|\|<br>3' AGUAGUAAUGGUCCGUCAUAAU | 5' ...UUGCAAUGAUGAAAGCAGCCUUU...<br>\| \|\| \|\|<br>3' AGUAGUAAUGGUCCGUCAUAAU |
| Position 5927-5933 of EGFR 3' UTR<br><br>hsa-miR-200b-3p | 5' ...GUUGAUUGUGCAUUGAGUAUUAA...<br>\| \|\| \|\| \|<br>3' AGUAGUAAUGGUCCGUCAUAAU | 5' ...GUUGAUUGUGCAUUGAGCCUUAA...<br>\| \| \| \|<br>3' AGUAGUAAUGGUCCGUCAUAAU |
| Position 4455-4462 of ETV1 3' UTR<br><br>hsa-miR-200b-3p | 5' ...UUUGCAUAUAGACUCCAGUAUUA...<br>\|\| \| \|\| \|\|<br>3' AGUAGUAAUGGUCCGUCAUAAU | 5' ...UUUGCAUAUAGACUCCAGGCUUA...<br>\|\| \| \|\|<br>3' AGUAGUAAUGGUCCGUCAUAAU |
